# Understanding, Experience, and Attitudes Toward Extended Reality Technology: A Multicenter Study

**DOI:** 10.1089/jmxr.2024.0002

**Published:** 2024-04-18

**Authors:** Jonathan R. Abbas, Oliver G. Townley, Azita Rajai, Antony Payton, Brendan A. McGrath, Neil Tolley, Iain A. Bruce, Rachel Isba

**Affiliations:** 1 Faculty of Biology, Medicine, and Human Health, The University of Manchester, Manchester, UK.; 2 Human Factors Academy, Manchester University NHS Foundation Trust, Manchester, UK.; 3 Stockport NHS Foundation Trust, Manchester, UK.; 4 Research and Innovation, Manchester University NHS Foundation Trust, Manchester Academic Health Science Centre, Manchester, UK.; 5 Centre for Biostatistics, Division of Population Health, University of Manchester, Manchester Academic Health Science Centre, Manchester, UK.; 6 Acute Intensive Care Unit, Wythenshawe Hospital, Manchester University NHS Foundation Trust, Manchester, UK.; 7 Imperial College Healthcare NHS Trust, London, UK.; 8 VREvo Ltd, Manchester, UK.; 9 Department of Paediatric Otolaryngology, Manchester University NHS Foundation Trust, Manchester, UK.; 10 Lancaster Medical School, Lancaster University, Lancaster UK.; 11 Alder Hey Children’s NHS Foundation Trust, Liverpool, UK.

**Keywords:** virtual reality, education, digital literacy, medical education

## Abstract

Extended reality technology (XRT) is predicted to play an important role in the future of health care. Although the hardware ecosystem is evolving rapidly, potential barriers to adoption include cost, physical space to use the hardware, side effects, and low understanding of the technology. This article aimed to explore these barriers by assessing the understanding, attitudes, and experience of junior doctors. In this multi-method, cross-sectional study, we administered a bespoke data capture tool to junior doctors in the North West of England. This focused on three domains: understanding, experience, and attitudes toward XRT in health care. Understanding was assessed by an objective knowledge test in a multiple-choice question format and specific self-assessed knowledge questions in a Likert-style questionnaire. Experience and attitudes toward XRT were measured using self-assessed experience questions and self-assessed attitude questions within the same Likert-style questionnaire. A total of 199/224 (89%) doctors who were approached participated in this study. The mean objective knowledge test score was 4.3/10 (range: 0.0–8.0; standard deviation = 1.7) and the median self-assessed knowledge questions score was 3.0/6.0 (interquartile range [IQR]: 2.0–4.0). The median self-assessed experience questions score was 2.2/6.0 (IQR: 1.5–3.5). In terms of attitude toward this technology, 185/199 (93.0%) of participants were interested in using this technology in medical education and similarly, 187/199 (94.0%) believed that it may be effective in medical training. This study demonstrated a low understanding of, and experience with, XRT in a population of junior doctors. Despite this, there was considerable interest in the potential value of this technology in health care, particularly within education. If XRT is to be widely adopted across the National Health Services, work is required to raise awareness of the technology, capabilities, and associated limitations.

## Introduction

Virtual reality (VR), augmented reality (AR), and mixed reality (MR) are all subtypes of extended reality technology (XRT), also known as “immersive technology.”^1^ XRT is predicted to play an increasingly prominent role in health care by academics, their institutions, and medical governing bodies.^1,2^ The SARS-CoV-2/COVID-19 pandemic, and prepandemic changes to trainees’ time and funding, have led to significant challenges in the education of both undergraduate medical students and the postgraduate medical workforce.^1^ With the rapid advancement in mobile computer processing, coupled with the paradigm shift toward remote, online learning, XRT has the potential to become commonplace within the medical curricula of the future.^1^

Simulation is commonly used across multiple industry sectors, and when combined with, or enhanced by XRT, has been used with great benefit, particularly in the aviation industry.^3^ XRT has the potential to enhance medical education for topics such as anatomy and physiology.^4^ In addition, it can provide a safe way for students to develop their procedural skills, with the complete elimination of risk to the patient.^4^ In the last 5 years, XRT has been featured in several landmark UK government reports, including *The Topol Review* and *The Future of Surgery: Technology Enhanced Surgical Training Report (FOS:TEST).*^5,6^ The FOS:TEST, published in 2022, provided an overview of technologies and how they may be used to enhance surgical training in the UK.^6^ Currently, the medical application of XRT is most prominent in the fields of remote surgical assistance, procedural step rehearsal, robotic surgery, and emergency triage readiness training.^6^ The Topol Review, written before the *FOS:TEST*, highlighted the importance of educating a more digitally literate health care workforce to foster innovation and improve patient care.^5^ At the time of its publication in 2019, the review predicted that immersive technology would have a steep upward curve of adoption and be widespread within the subsequent few decades.^5^

Although there is a rapidly progressing hardware ecosystem and a growing library of validated content, we do not understand the current rates of adoption of XRT by the medical workforce.^3^ This is of particular importance when planning the development of XRT tools for delivery at scale across the National Health Service (NHS).^3^ The terminology used throughout the literature to describe XRT can be confusing and has been criticized as being outdated potentially slowing adoption.^7,8^ Various other factors have been identified as important for the integration and uptake of XRT, including technology acceptance by the end-user, trialability, and cost.^9^

In the past 50 years, there has been significant research into the process of technology adoption. Central to this work was the influential book written by Rogers et al. entitled *The Diffusion of Innovation*, which then formed the basis of several subsequent technology adoption theories.^10,11^ The work initiated by Rogers and his group considered how an individual’s adoption of technology could lead to population diffusion, using the lenses of education, sociology geography, and a multitude of other factors.^9,10^ At their core, technology adoption theories are comprised of four key components; innovation, channels for communication, social systems, and time.^10^ The “digital age” has driven this research as technology is infiltrating all aspects of a doctor’s work, leisure, and social life.

This presented study aimed to establish the current baseline level of understanding of XRT among junior doctors. In addition, the levels of experience within this group were assessed while investigating any demographic or other individual factors, which may affect the current levels of experience and understanding of XRT. It is vital that technological development takes into account the specific educational needs of the user which cannot be decoupled from the users’ preconceptions and experiences.^12^

## Methods

### Study design and ethics

This study was a multi-method, cross-sectional, electronic survey-based research project conducted throughout NHS hospitals within the North West of England. Full prospective ethical approval was obtained from The University of Manchester (UoM) Research Ethics Committee (February 2, 2022; reference number: 2022–13152-21967). This study was then adopted onto the National Institute of Health and Care Research Clinical Research Network (NIHR CRN) portfolio on March 31, 2022.

### Study population

Junior doctors based in the North West of England, within their first 7 years of clinical practice, were invited to participate in this study. We aimed to capture the knowledge, experiences, and opinions of doctors working in a wide range of specialties with relatively undifferentiated career paths.

### Survey administration

To reduce the risk of non-response error, participants were recruited by one of the research team members (J.A.) during a regular group activity (e.g., face-to-face or online UK Foundation Programme or Core Surgical Training education days) within each of the postgraduate medical education centers within the North West of England. Administering the survey across multiple health care organizations ensured wider applicability of the study, sufficient participant numbers, and limited the chance of significant selection bias.^13^ One week before each session date, an electronic copy of the participant information sheet (PIS) was distributed from the program administrator to all attendees.

On the day of the session, a member of the research team reiterated the details in the PIS and displayed a QR code and weblink leading to the PIS, consent form, and Qualtrics^TM^ CoreXM survey for the participants to complete electronically, in real time.^14^ Each participant completed the survey once, which took approximately 12–15 min. If the session was face-to-face, an option to fill out the survey on article was also offered, in case of individual technical difficulties. If an article form was completed, responses were then inputted into Qualtrics^TM^ CoreXM by a member of the research team, following which the original article form was destroyed. One week later, each group was reminded of the survey and offered a final chance to take part. There was only a single follow-up communication facilitated by the program administrator, without any personal contact by the research team, to minimize any data protection or further ethical considerations.

### Survey design

The bespoke survey-based data collection tool was designed in conjunction with university educationalists and experts in knowledge assessment by multiple-choice questions (MCQ) alongside MCQ best practices (R.I. and T.P.). It was constructed and hosted online using the UoM-approved Qualtrics^TM^ CoreXM academic survey software. For additional clarity, a summary of outcome measures, and associated outcome measure instruments within the bespoke data collection tool is presented in Table 1. The survey consisted of the following four parts, which can be reviewed in full, in Supplemental Appendix:

**Table 1.** A Table to Delineate Which Specific Questions in the Bespoke Data Collection Tool Address Each Outcome Measure

Outcome measure	Outcome measure instrument	Specific questions of Bespoke Data Collection Tool, as numbered in Supplemental Appendix
Understanding of XRT	Objective knowledge test	1–10
	Self-assessed knowledge questions	16, 19, 22, 23
Experience with XRT	Self-assessed experience questions	14, 15, 17, 18, 20, 21, 24, 26
Attitudes toward XRT	Self-assessed attitude questions	11–13, 25
Tendency to engage with new technologies	Affinity for technology interaction scale^15^	27–35

1. **Demographic information (five questions)**—this information was used in the analysis to look for associations between individual factors, XRT experience, and understanding.
2. **XRT objective knowledge test (10 questions)**—these questions tested the participant’s understanding of XRT and consisted of a 10-question MCQ. Areas tested included discrimination between XRT sub-types, for example, use cases and matching the technology, and currently available hardware products.
3. **Self-assessed experience, knowledge, and attitude questions (16 questions)**—these questions explored the participants’ prior experience, perceived knowledge, and attitudes regarding XRT in medical education.
4. **Affinity for Technology Interaction [ATI] Scale (nine questions)—**this validated scale helped to define an individual’s “tendency to actively engage in intensive technology interaction.”^15^ This scale is an economical set of questions based on the Need for Cognition (NFC). The concept of NFC suggests that individuals widely differ in their willingness to engage in cognitive activities.^16^

**FIG. 1.**
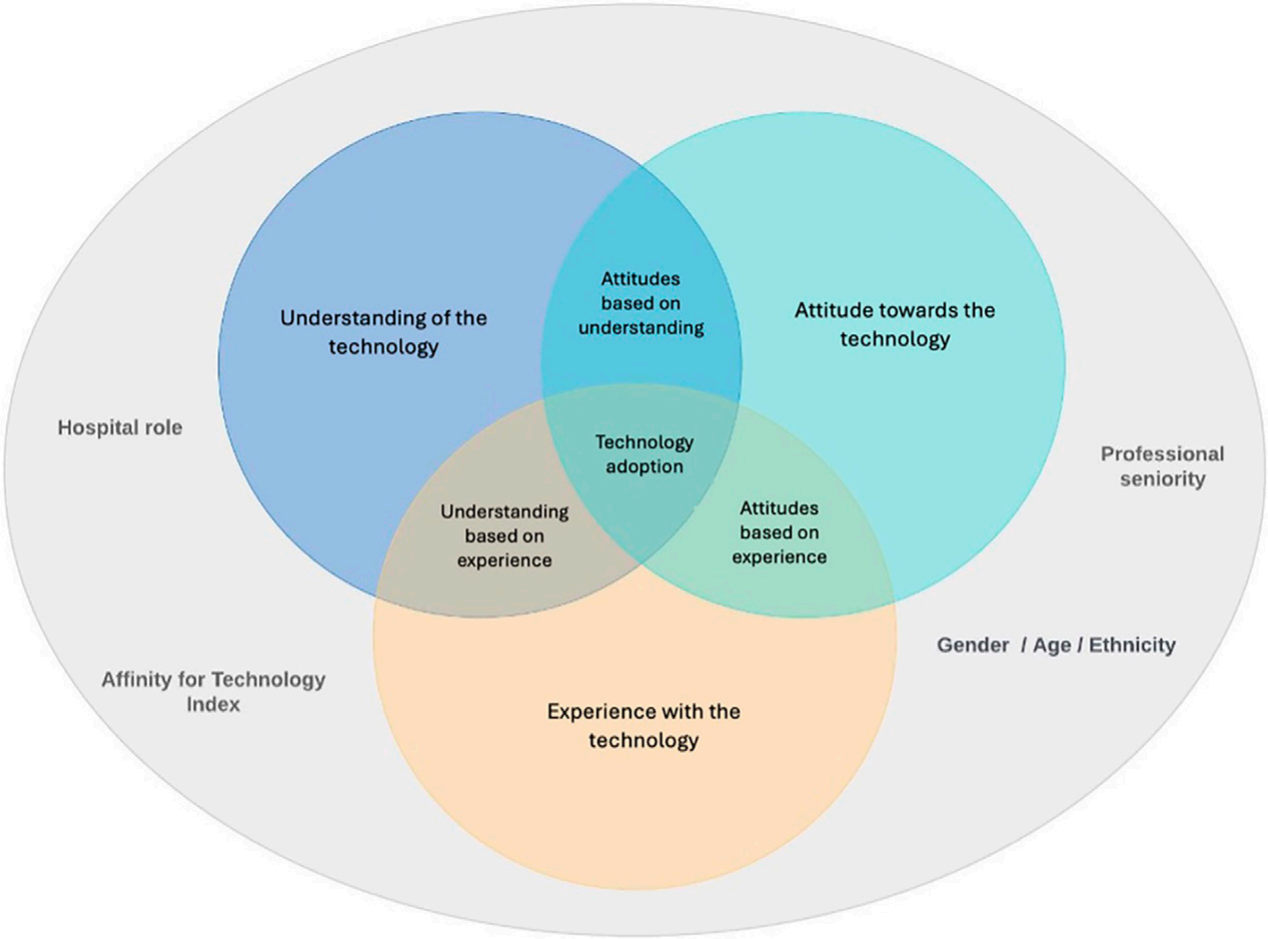
Diagrammatic representation of the overlap and interaction between the elements of the data collection tool. Domains such as experience, attitudes, and understanding of technology have a clear effect on the likelihood of technology adoption. The elements outside of the Venn diagram represent individual factors, which may affect any aspect of these domains.

A diagrammatic representation of how the above-described sections of the data collection tool interact with each other and contribute to overall technology adoption is presented in Figure 1.

### Data analysis

An *a priori* power calculation was performed using G*Power software 3.1. In order to identify a medium to low effect size for correlation (*d* = 0.4), with a power of 80% and an alpha error probability of 0.05, a sample size of 200 participants was required.^17^ Upon survey completion, distributions were explored with descriptive statistics and aggregate measure construction to include mean or median values as appropriate for the affinity for technology interaction (ATI) scale, objective knowledge test, and self-assessed experience, knowledge, and attitude questions depending on whether the data were parametric or non-parametric in nature. Results are represented as mean (standard deviation), median (inter-quartile range), or percentage agreement (95% confidence interval). Cronbach’s alpha testing was used to assess the internal validity of the questions within the self-assessed knowledge, experience, and attitude questions, as well as the ATI scale.

As a measure of the relationship between different participant factors, monotonic correlations were used with interpretation based on Mukaka’s 2012 description.^18^ For non-parametric distributions, Spearman rank correlation was used to assess the monotonic relationship. Statistical analyses were performed using R.4.1.2 (R Core Team).^19^

## Results

### Population characteristics

Between April 13, 2022 and July 19, 2022, 224 potential participants were approached who were present during a scheduled teaching activity, across five NHS hospital trusts in the North West of England. Of these, 199 junior doctors consented to and completed the survey leading to an overall participation rate of 89%. The included participants were based across five NHS trusts in the region. The majority of participants were working at the Foundation Programme level (internship program in the first 2 years postgraduation) or equivalent such as locally employed doctors at the same level of seniority (84.9%). We observed good representation across ages and genders and to a much lesser extent, ethnic groups. The full distribution of demographic data is presented in Table 2.

**Table 2.** Summary of Collated Participant Demographic Information Across the Study

Demographic factor	*n* = 199
Age [%]	
21–25	63 [31.7]
26–30	122 [61.3]
31–35	11 [5.5]
41–45	1 [0.5]
Prefer not to say	2 [1.0]
Gender [%]	
Female	111 [55.8]
Male	85 [42.7]
Prefer not to say	3 [1.5]
Ethnicity as defined by the UK government’s 2021 census categories [%]	
White (includes English, Welsh, Scottish, Northern Irish, British, Irish, Gypsy, Irish Traveler, Roma or any other White background)	139 [69.8]
Asian or Asian British (includes Indian, Pakistani, Bangladeshi, Chinese, or any other Asian background)	31 [15.6]
Black, Black British, Caribbean, or African (includes Black British, Caribbean, African, or any other Black background)	5 [2.5]
Mixed or Multiple ethnic groups (includes White and Black Caribbean, White and Black African, White, and Asian or any other Mixed or Multiple background-Asian/Asian British)	10 [5.0]
Other (includes Arab or any other ethnic group)*	9 [4.5]
Prefer not to say	5 [2.5]
Year of Graduation [%]	
2014	1 [0.5]
2015	1 [0.5]
2016	8 [4.0]
2017	16 [8.0]
2018	9 [4.5]
2019	4 [2.0]
2020	118 [59.3]
2021	41 [20.6]
Prefer not to say	1 [0.5]
Role [%]	
Foundation Trainee year 1 (or locally employed equivalent)	48 [24.1]
Foundation Trainee year 2 (or locally employed equivalent)	121 [60.8]
Core Surgical Trainee (or locally employed equivalent)	29 [14.6]
Other	1 [0.5]

This study surveyed a cross-sectional sample of junior doctors in the early years of their medical careers. In the North West of England, there are more than 7,000 junior doctors currently training, with a wide range of nationally and internationally diverse individuals represented in all areas of medicine.^20^ Although the data is not available to understand how many junior doctors, based in the North West of England are within the first 7 years of training, we can deduce that our study sampled at the very least around 3% of the total target population.

### Understanding objective knowledge test

The whole-group mean objective knowledge test score was 4.3 out of a maximum score of 10 (range 0.0–8.0; SD ±1.7). There was no significant difference between the objective knowledge test scores when grouping them by age or gender. Variations in scores were observed depending on the question subject matter, which can be seen in Table 3. Demonstrated here, knowledge of XRT hardware is least well demonstrated and knowledge of VR technology is most confidently shown. Objective knowledge test scores did not differ significantly between males and females (*p* = 0.11), or between age groups (*p* = 0.82).

**Table 3.** A Summary of the Knowledge Test and the Success Rate of the Participants Selecting the Correct Answer

Question number and type	Correct response % (*n* = 199)
1. Augmented reality technology identification	58.3
2. Virtual reality technology identification	74.9
3. Immersive technology hardware	10.6
4. Virtual reality definition	75.4
5. Mixed reality definition	57.8
6. Non-headset XRT	46.7
7. Mixed reality hardware	37.7
8. Immersive (360°) video	18.6
9. Immersive technology hardware identification	26.1
10. Adverse effects of XRT usage	21.6

XRT, extended reality technology.

### Understanding self-assessed knowledge questions

**FIG. 2.**
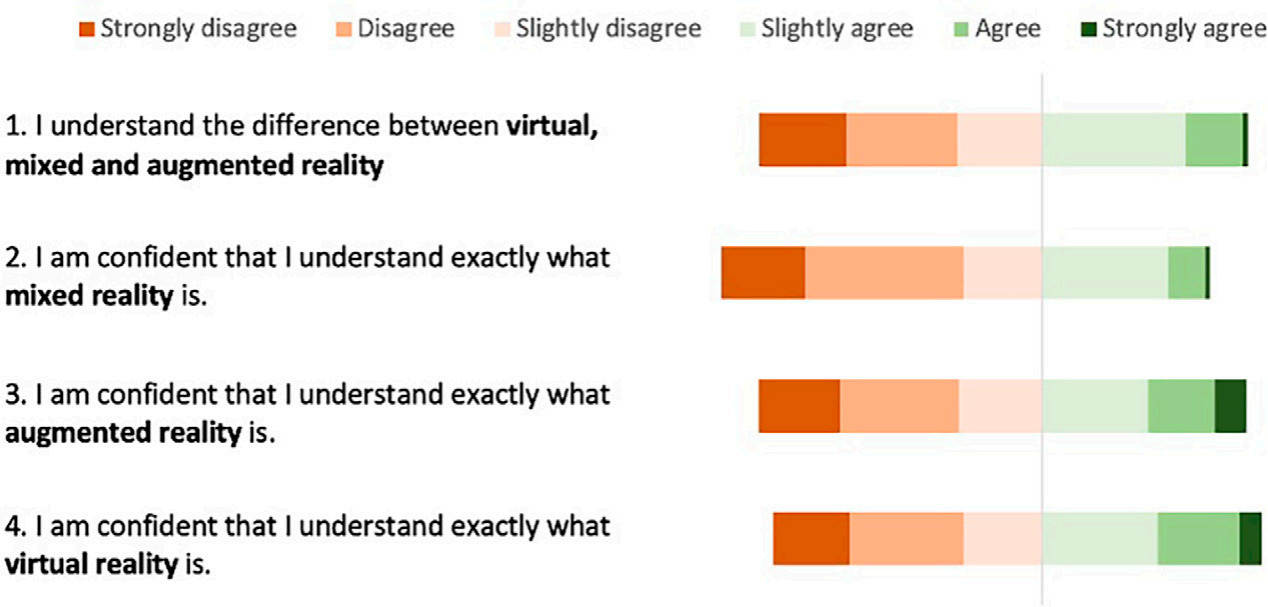
Summary of Likert responses for the self-assessed knowledge questions. The numbered statements are reproduced, in full, on the left with the Likert response rates visually displayed with each bar depicting the responses from the whole group. The focus of this was to determine familiarity and knowledge of XRT technology subtypes in bold. XRT, extended reality technology.

The 6-point Likert scale responses for the total self-assessed knowledge questions were converted into numerical values as a score out of six. The summary of the answers is presented in Figure 2. The median (IQR) score was 3.0 (2.0–4.0) with an associated Cronbach’s alpha was 0.91 (0.89–0.93) indicating an excellent measure of the reliability of questions.^21^ Higher median (IQR) self-assessed knowledge question scores were seen in male 3.3 (2.3–4.3) than female 2.8 (2.0–3.9) participants (*p* = 0.01) with no difference seen between age groups.

### Attitudes and experience—Self-assessed attitude and experience questions

**FIG. 3.**
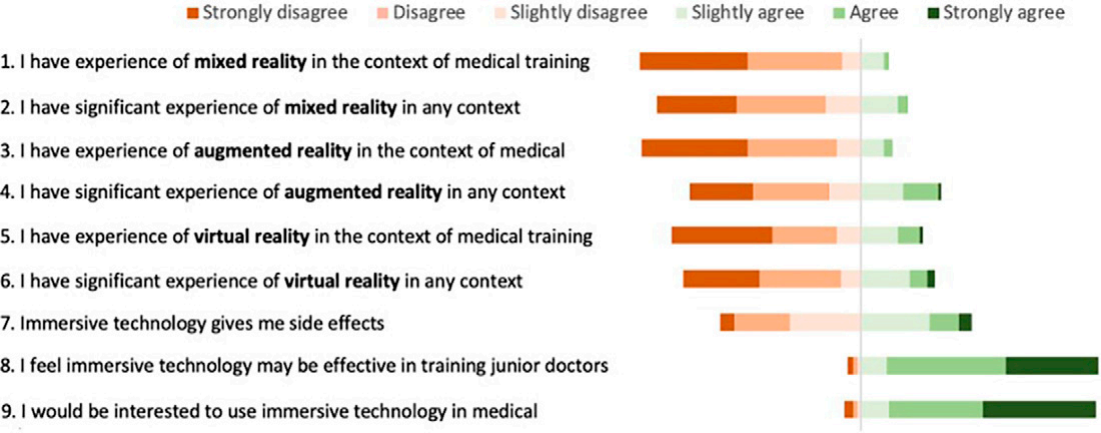
Summary of Likert responses to the self-assessed attitudes and experience questions, with XRT and the subtypes in bold. XRT, extended reality technology.

Figure 3 outlines a summary of the responses within the self-assessed experience and attitude scores. Questions labeled as 1–6 in Figure 3 are focused on gauging prior experience with XRT. When Likert responses were converted to a score out of six, the median (IQR) score for self-assessed experience was 2.2 (1.5–3.5). Cronbach’s alpha was calculated at 0.82 (0.77–0.85), indicating a high level of question reliability.

There was a weak correlation between gender and experience (*p* = 0.37), but scores were significantly higher in older trainees 3.2 (2.4–3.9) than the junior trainees 2.2 (1.5–2.7) (*p* = 0.01). Participants had significantly less experience with MR technology 2.0 (1.0–2.5) than both VR 2.0 (1.5–3.0), and AR 2.0 (1.5–3.0) technologies (*p* = 0.01).

Questions labeled 8 and 9 in Figure 3 enquire into the attitudes toward XRT in education. Overall, 185 (93.0%) of participants stated that they would be interested in utilizing the technology in health care and similarly 187 (94.0%) believed that it may be effective in training. When asked about perceived adverse effects (question 7 labeled in Figure 3) of the technology, 111 (55.8%) participants did not (or did not believe they would) experience side effects.

### Tendency to engage with new technologies-ATI scale

**FIG. 4.**
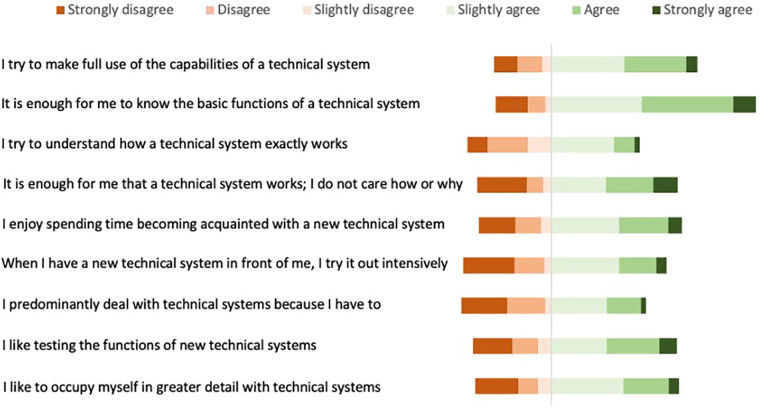
A visual summary of results from the ATI scale. Each bar demonstrates the whole cohort’s responses in order to proportionally visualize the spread of results. ATI, affinity for technology interaction.

Again, with an excellent level of question reliability, Cronbach’s alpha = 0.89 (0.86,0.91), and the mean (SD) whole group ATI scale was 3.53 (0.89). Figure 4 shows the results of the ATI scale by question. Several correlations have been calculated in order to identify any relationship between ATI scale and knowledge, and experience scores. There was a negligible correlation between the mean ATI score and objective knowledge (r = 0.21) and experience (r = 0.28). When correlating mean ATI scores with self-assessed knowledge scores, a low positive correlation was seen (r = 0.4).

## Discussion

This study demonstrated a low understanding of XRT among junior doctors in the North West of England. Although the evidence for the use of XRT within health care education, employee well-being, and patient care delivery is growing, this article is the first to investigate the uptake, attitudes, and barriers to the use of the technology among a population of junior doctors in the UK.^12,22^ Across many industries, technology literacy is fast becoming a core, mandatory skill. If adoption becomes ubiquitous, for example, as predicted by the Topol Review (2019), the requirement for XRT literacy may become a prerequisite of the health care workforce.^5,23^

### Understanding and experience of XRT is low

The presented results indicate low levels of understanding of the technology, both objectively and when participants self-assessed. Marks and Thomas describe prerequisites for mass adoption of XRT in education as trialability and technology acceptance.^4^ We would argue that before technology can progress from novelty to acceptable, the user base must have a firm understanding of its existence and at least a basic understanding of the capabilities presented. In addition, it is imperative that researchers investigating technology-enhanced education have a clear understanding of the technology being studied, validated, and experimented with. It has been suggested that the literature does not always describe technologies accurately which can in part, lead to confusion within the user-base and scientific community.^24–26^ There is, therefore, also a clear need to unify XRT nomenclature to reduce any future confusion in the context of research, design, and implementation.^24–26^

Similar to what is seen above in terms of understanding, self-assessed experience with XRT was also low. Although self-assessed experience question scores were low across all XRT subtypes (MR, VR, and AR), MR represented the lowest level of experience. Although XRT overall is gaining significant interest and beginning to be adopted, MR is a more recent technological advancement without the longer-standing technological history that we see for VR.^1,27^ Additionally, MR technology remains firmly within the realm of enterprise innovation, rendering it far less likely for an individual to have trialed it in their own personal life compared to consumer-focused VR solutions, which have seen recent popularization. With this in mind, there are numerous examples of MR being used within medical education including the enhancement of case-based teaching, anatomy demonstration, practical procedure tutorials, and remote ward rounds.^28–30^ Aside from technology novelty, we do not understand why the level of experience in this cohort was considerably low.

### Knowledge, experience, and individual participant factors

There was no discernible association between objective knowledge test or self-assessed knowledge question scores and individual demographic features of the participants. It is vitally important to understand that like technology adoption, opinion formulation around technology is complex. Not only does the device, solution, or software have to be fundamentally usable (with usability testing central to this), and fit for purpose, but individuals may require the satisfaction of many other factors.^31^ For example, an educator may look only to technology to *replace* existing teaching modalities in times of need (such as times of pandemic or war). Other individual educators or learners may look toward technology-enhanced educational tools as a way to *supplement* their established methods of learning. Most would require the technology to have a high degree of usability, comfort, and convenience although these factors would likely also vary between users.

In this study, we correlated the validated ATI scale with the user’s objective knowledge test scores, self-assessed knowledge question scores, and self-assessed experience question scores. The ATI scale is a “tool to quantify a key dimension of a users’ personality in the context of technology interaction.”^15^ By studying the above correlations, we begin to understand how an individual’s motivation to use novel technology may influence the likelihood of engagement or the level of positive attitude one may have toward XRT. Some correlation was observed when analyzing the ATI scale scores against the participants’ self-assessed knowledge. Given the novelty of XRT, this data suggests that the early adopters within the junior doctor cohort may have a propensity to seek out new, potentially complex technologies. The data presented in this study does not indicate a subgroup of junior doctors requiring more education around the technology, but that the group as a whole has little understanding of XRT.

When we consider the participants’ positive responses related to their attitudes toward XRT, we can see how these fit into the theories built upon the work conducted by Rogers.^10^ In stark contrast to the rest of the survey, the responses to these questions were overwhelmingly positive, demonstrating that junior doctors see potential value in XRT despite low levels of experience or understanding. For technology diffusion, the concept of “relative advantage” has emerged meaning that, for adoption to take place, the potential user base is required to understand and foresee value over existing solutions. As depicted in Figure 1, there are many factors that may influence technology adoption, some of which are measured in this article, demonstrating the complexity involved as a novel technology such as XRT begins to diffuse into the day-to-day working lives. The participants indicate a majority belief in the potential value it can bring indicating “relative advantage” over existing solutions. Straub builds on the notion suggesting the higher the relative advantage, the faster the potential adoption.^11^ We would importantly add that the NHS may not be the major driver of XRT adoption, but an organization, its staff, and patients that stand to benefit from this process.

## Strengths and limitations

This study provides valuable insights into the current understanding, experiences, and attitudes toward XRT in a cohort of junior doctors. The methodological strength is represented by its multisite design and low attrition rate. The study population included in this study may be representative across other seniorities and allied health care disciplines, and may also reflect the wider institutional experience, attitude, and understanding.

Limitations do however exist. Although the response rate was high, the overall participation rate as a percentage of the total population of junior doctors is low. In addition, the population of junior doctors was limited to the North West of England.

Second, we acknowledge that this study, due to its cross-sectional design, captures the understanding, opinions, and experiences of our participants at a single point in time. Although useful as a snapshot, longitudinal data, particularly after the implementation of an intervention would be an important area to focus on in the future.

Third, our methodological strategy was a fully quantitative approach. This approach has the potential to not capture the complexity and individual nuance around technology adoption and acceptability. We would recommend that future studies build upon this by conducting qualitative methods using focus groups or structured interviews in order to capture this important, yet absent data. Myriad individual factors can have an impact on the understanding of, experience with, and attitudes toward a particular technology.

## Recommendations and future directions

Sir Eric Topol identified several key disruptive technologies likely to significantly change the way health care is delivered in the future.^5^ In order to directly support this, government bodies have designed formal training within fellowships, accredited training programs, and endorsed higher educational activity around genomics, artificial intelligence, machine learning, and digital medicine.^5^ Currently running, wide-spread educational programs such as the Topol Digital Health Fellowship, Fellowship in Clinical Artificial Intelligence, and the Genomics Education Programme have been launched to arm clinicians with the tools to become literate in these emerging fields, have the ability to lead the implementation of these technologies in order to improve patient outcomes. Although XRT is cited alongside the above technologies, there are no such educational programs, fellowships or accredited education packages focused on educating the practicing front-line clinician in this area. We recommend that this clear gap in the educational provision by the NHS is addressed in order to educate the medical workforce appropriately. As recommended in the Topol Report, education of the workforce around the above digital technologies is focused on continual professional development activity for the entire workforce. Specialist training and the formulation of specialist digital technology career paths are reserved for those health care professionals with a focused interest. We recommend that this approach should be mirrored in the context of XRT in health care.

## Conclusion

With the rising interest and rapid development of XRT, clinicians, health care institutions, and government bodies are predicting prominence within health care in the future. Despite this, in a cohort of junior doctors, the level of understanding and experience of XRT is low, while the same group indicated overwhelmingly positive attitudes toward the potential health care benefit of this technology. These findings may indicate a significant barrier to the adoption of potentially positive solutions in health care education, therapy, and wellness. As highlighted by several landmark UK government commissions, we are likely to see an increase in the development and adoption of education and therapeutic XRT tools. For these to be widely adopted and the potential benefit to staff, patients, and organizations to be realized, it is important that the medical workforce is appropriately educated in order to increase the overall immersive technology digital literacy.

## Abbreviations Used

XRT: Extended reality technology
VR: Virtual reality
AR: Augmented reality
MR: Mixed reality
FOS:TEST: The Future of Surgery: Technology Enhanced Surgical Training Report
NHS: National Health Service
UoM: The University of Manchester
NIHR CRN: National Institute of Health and Care Research Clinical Research Network
PIS: Participant Information Sheet
MCQ: Multiple-choice questions
ATI: Affinity for Technology Interaction
NFC: Need for Cognition
